# Consecutive Inhibition of Telomerase and Alternative Lengthening Pathway Promotes Hodgkin’s Lymphoma Cell Death

**DOI:** 10.3390/biomedicines10092299

**Published:** 2022-09-16

**Authors:** Matheus Fabiao de Lima, Monique Oliveira Freitas, Mohammad K. Hamedani, Aline Rangel-Pozzo, Xu-Dong Zhu, Sabine Mai

**Affiliations:** 1 Department of Physiology and Pathophysiology, CancerCare Manitoba Research Institute, University of Manitoba, Winnipeg, MB R3E 0V9, Canada; 2 Genetic Service, Institute of Paediatrics and Puericulture Martagão Gesteira (IPPMG), Federal University of Rio de Janeiro (UFRJ), Rio de Janeiro 21941-912, Brazil; 3 Department of Biology, Faculty of Science, McMaster University, Hamilton, ON L8S 4K1, Canada

**Keywords:** telomerase, alternative lengthening of telomeres (ALT), BIBR 1532, trabectedin, promyelocytic leukemia bodies (PML), telomeric repeat-binding factor 1 (TRF1)

## Abstract

Telomere maintenance is key during cancer development. Malignant cells can either use telomerase or an alternative lengthening of telomere (ALT) pathway to maintain their telomere length. In Hodgkin’s Lymphoma (HL), the presence of telomerase activation is established. The activation of ALT has been reported recently. Our data confirm this notion describing co-localization of the phosphorylated form of telomeric repeat-binding factor 1 (pT371-TRF1) with ALT-associated promyelocytic leukemia bodies. Surprisingly, to our knowledge, there are no published studies targeting both telomere maintenance pathways in HL. Consequently, we investigated, for the first time, the effects of both telomerase and ALT inhibition on HL cell viability: We inhibited telomerase and/or ALT, given either individually, simultaneously, or consecutively. We report that the inhibition of telomerase using BIBR1532 followed by ALT inhibition, using trabectedin, caused a decrease of greater than 90% in cell viability in three patient-derived HL cell lines. Our results suggest that HL cells are most vulnerable to the consecutive inhibition of telomerase followed by ALT inhibition.

## 1. Introduction

Hodgkin’s lymphoma (HL) is a B-cell malignancy characterized by the presence of mononucleated Hodgkin (H) and multinucleated Reed–Sternberg (RS) cells [1,2], with the RS cell representing the diagnostic cell of the disease [2]. In 2020, more than 83,000 people globally were diagnosed with classical Hodgkin’s Lymphoma (cHL), and more than 23,000 deaths were registered for the same period [3]. HL can affect all age groups [4,5]. There are high cure rates for cHL when diagnosed at early disease stages or in patients presenting with low-risk disease [4,6]. However, patients diagnosed at advanced disease stage have poor prognosis and unfavorable disease outcome [6]. The 5-year relative survival rate is 89.1% (seer.cancer.gov).

Telomeres are protective heterochromatic structures found at the ends of linear eukaryotic chromosomes. Mammalian telomeric DNA is composed of repetitive TTAGGG tandem repeats. The number of times most normal human somatic cells can divide is linked to the status of their telomeres. Telomeric DNA shortens during each round of DNA replication, in part because DNA polymerase is unable to completely replicate the 3′ overhang of the chromosomal ends (the “end replication problem”) [7,8,9,10]. Once telomeric DNA reaches a critical short length, the cells stop dividing and enter a state called senescence. This state of a limited life span was first defined by Hayflick [11,12] and it has also been coined as the Hayflick limit.

In stark contrast to normal cells, tumor cells escape the Hayflick limit of lineage-dependent limited life span. This step involves mutations inactivating cell cycle checkpoint proteins, increased cell divisions and therefore further telomere shortening. These events trigger a “crisis” event where sister chromatin fusions, end-to-end fusions and breakage-bridge-fusion cycles events occur, leading to chromosomal rearrangements and ongoing genome instability. While most cells die during crisis, some survive and activate telomere maintenance mechanisms. These telomere maintenance pathways permit the cells to continue cellular division despite the presence of critically short telomeres. There are two telomere maintenance pathways, telomerase activation and alternative lengthening of telomeres (ALT) [13,14,15]. Telomerase activation is the most common pathway and is employed by 85–90% of all tumor cells, while ALT is used by the remainder. Some tumors, albeit rare, show the co-activation of both pathways. These include rare cases of renal cell carcinoma [16], breast cancer [17] and classical Hodgkin’s Lymphoma [18]. The consequence of continued cell proliferation of tumor cells despite their critically short telomeres allows for ongoing genomic instability and its dynamic propagation to the next generation of cells.

Genomic instability is an enabling hallmark of cancer [19]. The contribution of telomeres to this dynamic process of genomic instability is genomic remodeling through sister chromatid fusion, end-to-end chromosomal fusion, and breakage-bridge-fusion cycles [20,21]. This dynamic process of telomeric changes is also called telomere dysfunction and is a key factor in creating cell-to-cell genetic heterogeneity in cancer and enables tumor cell and clonal evolution [22]: For example, a single telomeric end-to-end chromosomal fusion will generate, following cell division, due to the formation of an anaphase bridge and chromosomal breakage in telophase, one daughter cell with a terminal deletion and one with an unbalanced translocation. Both daughter cells are then genetically distinct and due to the presence of double-strand breaks at the chromosomal break points, they will engage in further fusion/recombination events with other chromosomes. This process will continue and lead to the genetic divergence of the cells and, upon selective pressure, to the generation and survival of clones [22,23].

Hodgkin’s Lymphoma exhibits telomere dysfunction [24], telomerase activation [24,25] and ALT activation [18]. Since tumor cells evolve to become resistant and overcome telomerase inhibition by activating the ALT pathway [26], it is expected that the dual inhibition of both pathways will be more effective than the single pathway inhibition. While the single inhibition of one of the two telomere maintenance pathways has been reported [27,28,29], there is no published work to date demonstrating the dual inhibition of both pathways. Telomerase inhibition is effective in telomerase-activated cancer cells [30,31]. However, as a result of this treatment, the cells commonly become resistant [26]. Inhibition of ALT is currently offered to patients with soft tissue sarcoma [32] and ovarian cancer [33].

The degree of telomere dysfunction in Hodgkin’s Lymphoma [24] is associated with the aggressiveness of disease and response to treatment [34,35]. As we showed, telomere dysfunction (measured by three-dimensional (3D) telomere imaging and dedicated software) in mononucleated Hodgkin’s cells leads to the formation of the diagnostic Reed–Sternberg cell [2,24,36]. Telomere dysfunction in HL includes critically short telomeres, telomeric aggregates, altered telomere numbers and a different 3D spatial organization [24,34]. In addition, the severity of the 3D telomere dysfunction in HL is associated with response to treatment and recurrent/refractory disease [24]. The higher the level of telomere dysfunction, the greater the chance of poor outcome [34,35,36].

The current study was initiated to explore the addiction of Hodgkin’s Lymphoma cells to telomere maintenance. Using the small molecule inhibitor BIBR1532 to inhibit telomerase [37,38] and the alkylating agent trabectedin to inhibit the ALT pathway [27] alone and in combination, as well as with a consecutive addition of either drug, we report that telomerase inhibition for 72 h, followed by ALT inhibition for 72 h exhibits the strongest negative effects on cell viability of HL cells.

## 2. Materials and Methods

### 2.1. Cell Lines and Cell Culture Conditions

Three different human-derived HL cell lines were used for this study: HDLM-2, L-428 and L-1236 (DSMZ, Braunschweig, Germany). The HDLM-2 and L-428 cell lines were grown in RPMI-1640 medium, supplemented with 20% fetal bovine serum (FBS), 1% L-glutamine, 1% sodium pyruvate, and 1% penicillin–streptomycin (reagents from Invitrogen/Gibco, Burlington, ON, Canada). The L-1236 cell line was grown in RPMI-1640 medium, supplemented with 10% FBS, 1% L-glutamine, 1% sodium pyruvate, and 1% penicillin–streptomycin. Cells were incubated at 37 °C in a humidified atmosphere containing 5% CO_2_. A concentration of 5 × 10^6^ cells/tissue culture well in 6-well plates (Nunc^TM^ Cell Culture Treated Multidishes, ThermoFisher Scientific, Waltham, MA, USA) were used for all cell lines during the inhibition of telomere maintenance pathways assay. This cell number was chosen to simulate the overall number of lymphocytes residing in a regular lymph node [39].

### 2.2. Fluorescent Immunocytochemistry

#### 2.2.1. Antibodies

Antibodies used include: primary anti-TRF2 (rabbit polyclonal, Novus (NB110 57130) 1:500 dilution); secondary goat anti-rabbit Alexa 488 (Molecular Probes, Waltham, MA, USA, 1:1000 dilution); primary anti-pT371-TRF1 [40] (rabbit polyclonal, 1:500 dilution); primary anti-PML (mouse monoclonal, Santa Cruz, sc- 966, 1:100 dilution), and sheep anti-mouse Cy3 (AC111C, Sigma Chemical, St. Louis, MO, USA, 1:500 dilution). The antibodies used for immunocytochemistry analyses were diluted in 4% BSA/4X SSC (blocking solution).

#### 2.2.2. Immunocytochemistry

The cells were fixed in 3.7% formaldehyde/1× PBS for 10 min at room temperature (RT) and immunostained as previously published [24,36]. Cell nuclei were counterstained with DAPI 0.1 µg/mL (D9542, Sigma Chemical, St. Louis, MO, USA) for 3 min. The slides were mounted in Vectashield^®^ (Vector Laboratories, Inc., Burlingame, CA, USA).

#### 2.2.3. Three-Dimensional Image Acquisition

Three-dimensional conventional imaging of 90 cells from each cell line was performed using a ZEISS Axio Imager Z2 (Carl Zeiss, Toronto, ON, Canada) with a cooled AxioCam HR B&W, FITC, Cy3 and DAPI filters in combination with a Planapo 63×/1.4 oil objective lens (Carl Zeiss, Jena, Germany). For every fluorophore, 60 z-stacks were imaged with a 200 nm distance between each stack. Images were obtained using ZEN blue 2.3 edition software (Carl Zeiss, Jena, Germany) in multichannel mode, and deconvolved using the constrained iterative restoration algorithm [41] with theoretical PSF and automatic normalization.

### 2.3. Inhibition of Telomere Maintenance Pathways

The initial drug concentrations were selected based on prior studies reporting the inhibition of ALT and telomerase pathways in different cancer cell lines [27,42]. To define the drug working concentration, the HL cell line HDLM-2 was subjected to individual treatment with telomerase inhibitor BIBR1532 (EMD MilliporeSigma, San Luis, MO, USA) using the following concentrations: 125, 150, 175, 200 µM from 0–144 h and treatment with ALT-pathway inhibitor trabectedin (Apexbio Technology, Houston, TX, USA) using the following concentrations: 0.25, 0.5, 0.75, 1, 1.25 nM from 0–240 h [27]. The concentrations determined with HDLM-2 were applied to the other HL cell lines L-1236 and L-428.

### 2.4. Cell Viability

**Figure 1:**
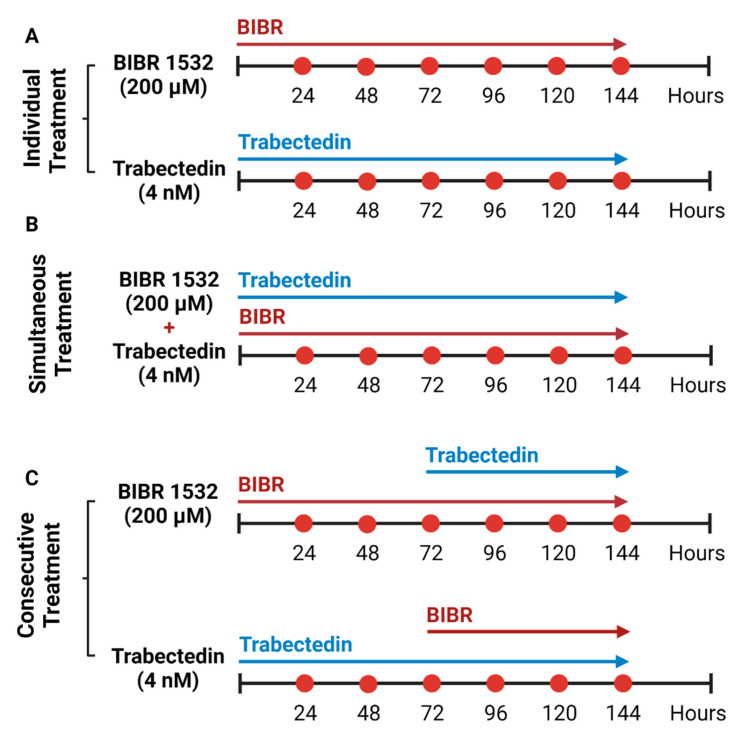
Inhibition of telomere maintenance pathways in HL cell lines. (**A**) Individual treatment of HL cell lines with 200 µM of BIBR1532 and 4 nM of trabectedin for 144 h. (**B**) Simultaneous treatment of HL cell lines with 200 µM of BIBR1532 and 4 nM of trabectedin for 144 h. (**C**) Consecutive treatment of HL cell lines with BIBR1532 and trabectedin treatment, the latter was added after the first 72 h of BIBR1532. Consecutive treatment of HL cell lines with trabectedin followed by BIBR 1532, which was added at the 72 h time point.

**Figure A1:**
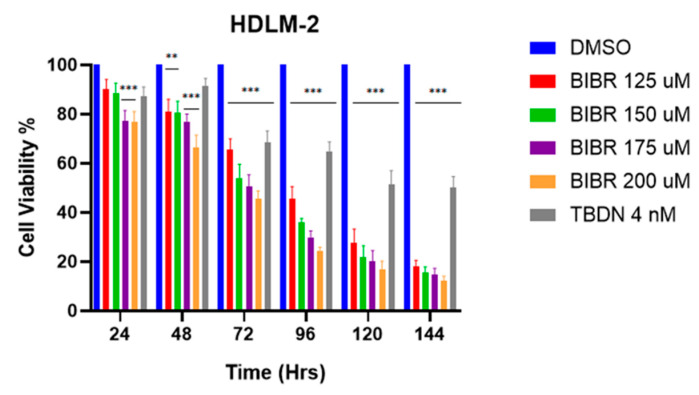
BIBR1532 concentration titration using HDLM-2 cell line. Decrease in cell viability upon increase in BIBR1532 concentration. The decrease in viability is time-dependent as well as dose-dependent. Data expressed as mean ± SEM. ** *p* < 0.01 and *** *p* < 0.001. TBDN—trabectedin.

The HL-derived cell lines (HDLM-2, L-428 and L-1236) were seeded in 6-well plates (Nunc^TM^ Cell Culture Treated Multidishes, ThermoFisher Scientific, Waltham, MA, USA) at 5 × 10^6^/well. The cells were treated with DMSO to a final concentration of 0.02% (control condition), 200 µM of telomerase-inhibitor BIBR1532 and 4 nM of ALT-pathway inhibitor trabectedin in various combinations and orders as shown in Figure 1. The trabectedin and BIBR 1532 treatment concentrations were determined using the HDLM-2 cell line (Figure A1).

The cell viability before and during treatments was evaluated by trypan-blue exclusion assay every 24 h. Briefly, aliquots containing 15 µL of the cells were taken every 24 h, mixed with 15 µL of trypan blue (1:1 ratio) and submitted (11 µL) to manual counting using a hemocytometer (Hausser Scientific, Horsham, PA, USA).

### 2.5. Statistical Analysis

Two-way ANOVA followed by Tukey’s test were used to analyze the impact on cellular viability of the HL cell lines (HDLM-2, L-428 and L-1236) treated with telomerase and ALT inhibitors. Significance levels were set as *p* ≤ 0.05. PRISM Graph Pad v8.0 (San Diego, CA, USA) software was used for illustration.

## 3. Results

### 3.1. Hodgkin’s Lymphoma (HL) Shows Hallmarks of an Alternative Telomere Lengthening (ALT) Pathway

As previously reported by us and others, HL cells activate telomerase [24,25]. Recent work described the alternative lengthening of telomeres (ALT) pathway, which involved the presence of promyelocytic leukemia (PML) bodies [18,43]. Since PML bodies can be found in non-malignant cells [44], we examined the presence of both PML and pT371-TRF1 protein in HL cells. The latter does not bind normal telomeres [40] but interacts specifically with dysfunctional telomeres, forming distinct foci in ALT cells [45]. In addition, pT371-TRF1 is a component of APBs, a hallmark of ALT [45].

**Figure 2:**
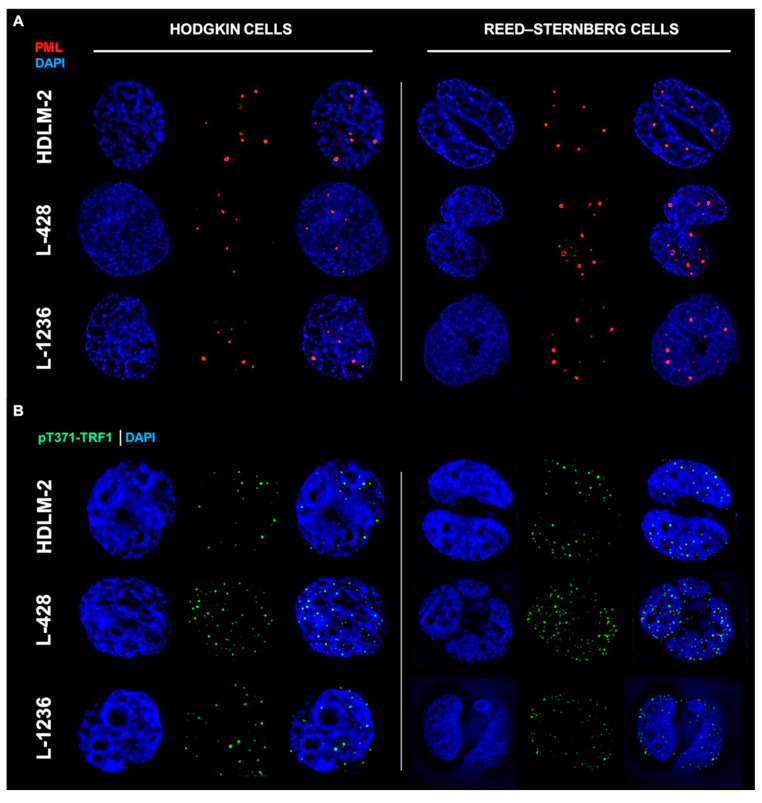

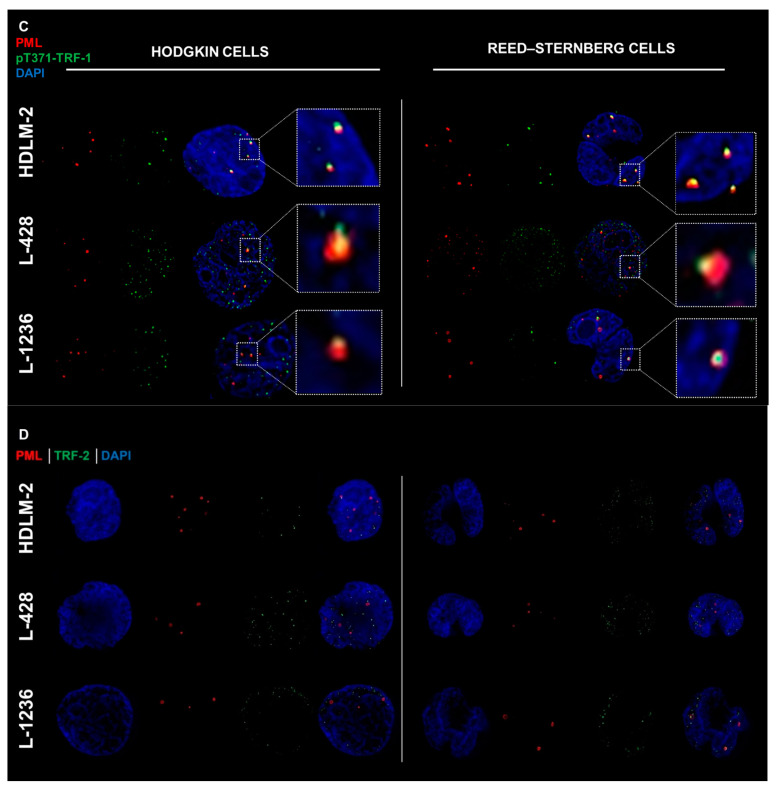
Hodgkin’s Lymphoma (HL) shows hallmarks of an alternative telomere lengthening (ALT) pathway. (**A**) HL cell lines show promyelocytic leukemia bodies (PML) in both HL cell phenotypes (Hodgkin cells and Reed–Sternberg cells). (**B**) HL cell lines show phosphorylated form of telomeric repeat-binding factor 1 (pT371-TRF1) in both HL cell phenotypes. (**C**) HL cell lines show colocalization (white zoom boxes) of pT371-TRF1 and PML bodies in both HL cell phenotypes. (**D**) HL cell lines show presence of telomeric repeat-binding factor 2 (TRF2) around PML bodies in both HL cell phenotypes. PML—Red (Cy3), pT371-TRF1—Green (A-488), TRF2—Green (A-488) and DNA—Blue (DAPI). A total of 90 cells were examined in three independent experiments for each of the HL cell lines, and all showed both colocalized (yellow) as well as free signals for pT371-TRF1 and PML in addition to TRF2 and PML in both H and RS cells. For additional information, see Appendix A Figure A2.

**Figure A2:**
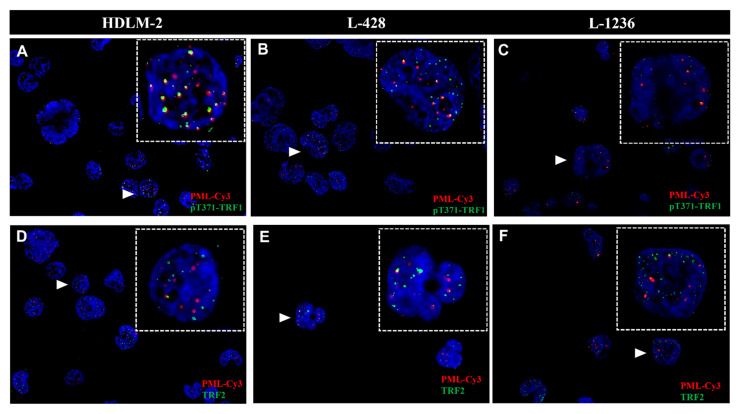
Hodgkin’s Lymphoma shows ALT activation hallmarks. Analysis of colocalization of PML bodies, pT371-TRF1 and TRF2 in Hodgkin’s Lymphoma cell lines (HDLM-2, L-428 and, L-1236). (**A**) Analysis of colocalization of PML bodies and pT371-TRF1 in HDLM-2. A white arrow shows the zoomed cell. (**B**) Analysis of colocalization of PML bodies and pT371-TRF1 in L-248. A white arrow shows the zoomed cell. (**C**) Analysis of colocalization of PML bodies and pT371-TRF1 in L-1236. A white arrow shows the zoomed cell. (**D**) Analysis of colocalization of PML bodies and TRF2 in HDLM-2. A white arrow shows the zoomed cell. (**E**) Analysis of colocalization of PML bodies and TRF2 in L-428. A white arrow shows the zoomed cell. (**F**) Analysis of colocalization of PML bodies and TRF2 in L-1236. A white arrow shows the zoomed cell. Colocalized signals are shown in yellow. PML (Cy3-Red). pT371-TRF1 (FIT C-Green). TRF2 (FITC-Green). 90 cells were examined in three independent experiments and for each cell line. All cell lines display colocalization of pT371-TRF1 with PML and TRF2 with PML. However not all signals in each cell colocalize.

We performed immunofluorescent analysis of PML and pTRF1 in three HL cell lines (HDLM-2, L-1236 and L-428). First, the presence of PML bodies was confirmed in all HL cell lines, and observed in both mononucleated Hodgkin’s (H) cells and multinucleated Reed–Sternberg (RS) cells as shown in Figure 2A. The presence of the phosphorylated form of the telomeric repeat-binding factor 1 (pT371-TRF1) was also confirmed in all HL cell lines, in both H and RS cells, as shown in Figure 2B. In addition, colocalization of pT371-TRF1 and PML bodies was observed in all HL cell lines (Figure 2C and Appendix A, Figure A2), suggesting the presence of APBs, hallmarks of ALT, in all HL cell lines.

The telomeric repeat-binding factor 2 (TRF2) protein is associated with ALT telomere synthesis and works to maintain ALT pathway activity [46,47]. TRF2 protein is seen in all HL cell lines and, in both H and RS cells (Figure 2D). APBs are present in all HL cells.

Analysis of colocalization to pT371-TRF1 and PML as well as of TRF2 and PML in 90 cells and three independent experiments showed for each of the HL cell lines that each cell exhibited both colocalized and free signals of pT371-TFR1/PML and TRF2/PML (Figure 2C,D). The reasons for the heterogeneous signal localization are currently unknown.

### 3.2. HL Is Sensitive to the Inhibition of Both Telomere Pathways

We hypothesized that the inhibition of both telomere maintenance pathways might be lethal to HL cells. We therefore treated the HL cells with telomerase inhibitor alone, ALT inhibitor alone, both telomerase and ALT inhibitors at the same time or sequentially.

### 3.3. Independent and Simultaneous Inhibition of Telomere Maintenance Pathways

**Figure 3:**
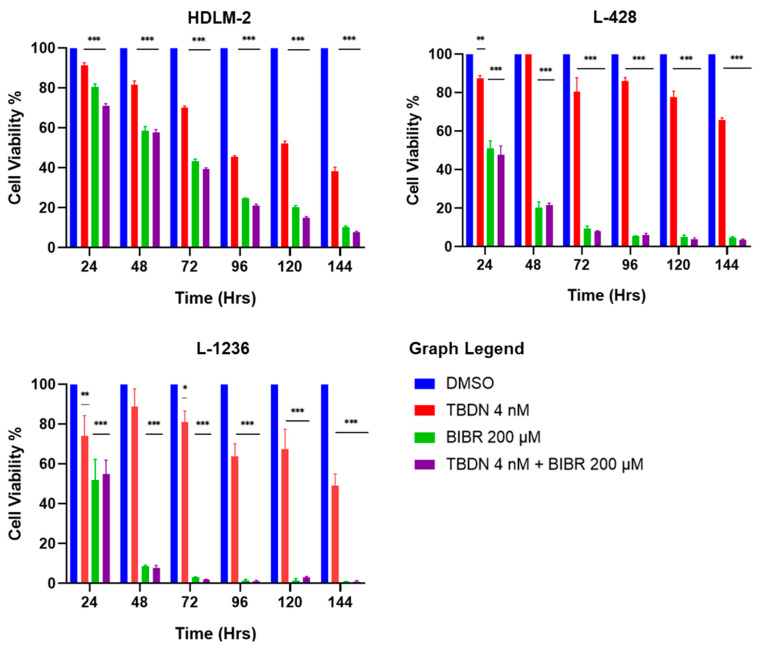
Combinatory inhibition of telomere maintenance pathways. HL cell lines show decrease in cellular viability upon treatment with trabectedin 4 nM (ALT pathway inhibitor). The effect was time-dependent in all HL cell lines. Incubation of HL cell lines with 200 µM BIBR 1532 (telomerase inhibitor) showed significant time-dependent decrease in cell viability. The combined inhibitory effect on both telomere maintenance pathways resulted in a remarkable time-dependent decrease in cellular viability, in all HL cell lines. Data expressed as mean ± SEM. * *p* < 0.05, ** *p* < 0.001 and *** *p* < 0.0001. TBDN—trabectedin.

Exposure of HDLM-2, L-428 and L-1236 HL cell lines to 4 nM of trabectedin [27] (ALT pathway inhibitor) or 200 µM of BIBR1532 (telomerase inhibitor) [48] for 144 h led to a time-dependent decrease in cell viability. As shown in Figure 3, all three cell lines exhibited a reduction in cell viability. At 144 h, trabectedin triggered 60%, 40% and 50% survival of HDLM-2, L-428 and L-1236, respectively. BIBR1532 reduced cell viability by ≥ 90% in all three cell lines during the same period. The simultaneous treatment with both drugs elicited survival similar to the BIBR1532 treatment regimen alone (Figure 3).

### 3.4. Consecutive Inhibition of Telomere Maintenance Pathways

**Figure 4:**
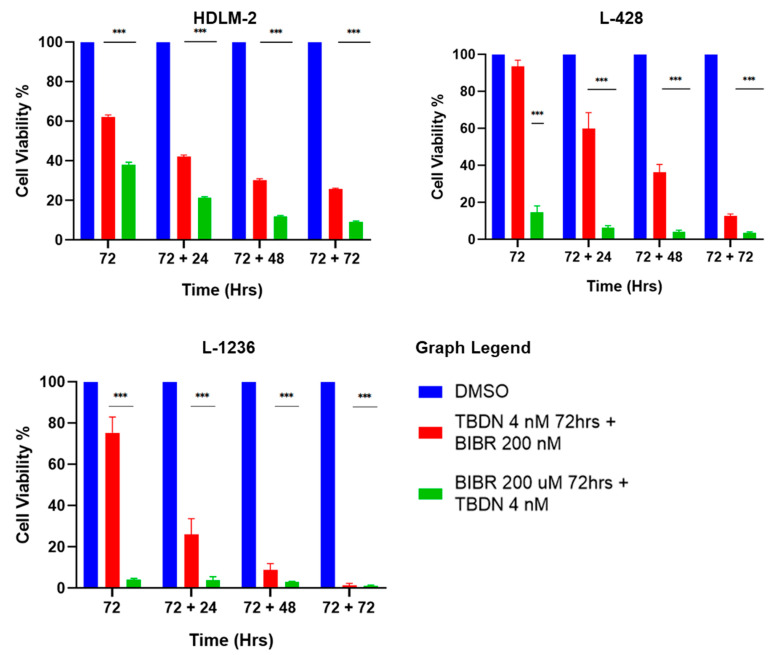
Consecutive inhibition of telomere maintenance pathways. HL cell lines show pronounced reduction in cellular viability over cell sensitization with trabectedin 4 nM and BIBR 200 µM for 72 h, followed by consecutive treatment with trabectedin 4 nM and BIBR 200 µM for 72 h. Data expressed as mean ± SEM. *** *p* < 0.0001. TBDN—trabectedin.

The consecutive exposure of HDLM-2, L-428 and L-1236 to trabectedin and BIBR1532 for 72 h each (Figure 1) was performed to determine the effects of dual but consecutive telomere maintenance pathway inhibition on HL cells. Trabectedin treatment followed by BIBR1352 treatment induced cell death at 75%, 90% and >95% in HDLM-2, L428 and L-1236, respectively. This effect was stronger than that of trabectedin alone (Figure 4). However, the consecutive treatment of BIBR1352, followed by trabectedin decreased cell survival to 10%, <5% and <5% in HDLM2, L-428 and L-1236, respectively. This consecutive treatment order reduced the viability of HL most dramatically. Collectively, our results indicate that telomerase inhibition followed by ALT inhibition leads to the most potent induction of HL cell death.

## 4. Discussion

Telomerase activation is key to maintaining an unlimited proliferation potential, which is characteristic of malignant cells [49,50]. However, not all tumor cells express telomerase. Some tumor cells instead activate an alternative pathway for lengthening of telomeres (ALT) and, in some cases, malignant cells activate both pathways [14,49,51,52,53]. In Hodgkin’s Lymphoma, the presence of telomere maintenance pathways through telomerase and ALT was reported for human-derived lymph node samples as well as HL cell lines [18,24,25]. However, only promyelocytic leukemia bodies (PML) were investigated as an ALT marker, and a high frequency of cell-to-cell heterogeneity was observed [18]. Moreover, the presence of PML proteins is also observed in non-malignant cells [44], highlighting the need for further characterization of ALT activation in HL.

Here, we show the presence of ALT-associated promyelocytic leukemia bodies (APBs), TRF2, pT371-TRF1 and for the first time the colocalization of pT371-TRF1 and PML in three patient-derived Hodgkin lymphoma cell lines in both HL and RS cells. Association of TRF2 and APBs are hallmarks of ALT activation in HL [18,53].

The phosphorylation of TRF1 protein on T371 residue promotes its interaction with APBs, therefore causing activation of ALT [45]. Moreover, loss of TRF1 phosphorylation or TRF1 deletion impairs formation of APBs, disrupting ALT pathway activity [45,54]. Here, we show the presence and colocalization of pT371-TRF1 with APBs in HL cells and confirm ALT activation in HL and RS cells of HL.

The activation of telomere maintenance pathways has been correlated with malignant transformation and cancer progression and is also associated with poor prognosis and reduced overall survival [55,56,57,58]. The characterization of telomere maintenance pathways has been used to assess cancer aggressiveness and stratify malignant subgroups [27,56,58]. Although several studies have been targeting telomere maintenance pathways in cancer cells, these inhibitors are not currently used to treat HL in clinical practice [27,32,59,60,61]. Importantly, targeting telomerase alone was shown to induce activation of ALT in cancer cells [26,62], highlighting the need for new therapeutical approaches that target telomerase in addition to ALT.

Here, we show that consecutively targeting telomerase and the ALT pathway induces cell death in HL cell lines synergistically by >90%. Recent studies proposed trabectedin as a co-adjuvant drug to treat classical Hodgkin’s Lymphoma, due to its anti-tumoral activity and tumor microenvironment modulatory ability [28,29]. Additionally, recent clinical trials have explored the potential use of trabectedin, due to its high efficacy and fewer side effects, compared to standard cancer treatments [63,64,65,66,67,68]. Others have reported trabectedin as an effective anti-tumoral drug against advanced soft tissue sarcomas and ovarian cancer in clinical settings [69,70,71,72].

BIBR1532 has been used in several studies as a specific and powerful telomerase inhibitor [48,73,74,75]. It demonstrates anti-migration and anti-proliferation properties in addition to its cytotoxic effect on cancer cells [76,77]. Recent clinical trials using a different telomerase inhibitor (Imetelstat) showed promising results against multiple myeloma, myelodysplasia, acute myeloid leukemia, and myelofibrosis, even in patients presenting resistance to first-line treatment options [78,79,80,81,82,83]. This highlights the potential use of telomerase inhibitors as effective drugs in the treatment of hematological cancers.

Although most cancers activate one of the two telomere maintenance pathways, targeting just one was shown to induce the activation of the other [26]. Here, we highlight that the consecutive use of telomerase inhibitor (BIBR1532) followed by ALT inhibitor (trabectedin) within 72 h is necessary to achieve a high impact on HL cell viability (>90% decrease in cell viability) compared to cell death induced by each inhibitor alone or by combined ALT and telomerase inhibition and consecutive inhibition of first ALT and then telomerase. A limitation of our study is the use of patient-derived cell lines. Future investigations of primary pre-treatment HL lymph node aspirates will be required. The ex vivo treatment of such primary HL cells with the inhibitors of both telomerase maintenance pathways will be absolutely essential prior to any future clinical trials and a potential translation to clinical application. Moreover, future translational studies will determine whether the inhibition of telomere maintenance pathways (alone or in combination with other treatments) could be used as novel therapeutical avenues to treat hematological and solid cancers.

## 5. Conclusions

Hodgkin’s Lymphoma cells exhibit telomerase and alternative telomere lengthening pathways. The present study investigates whether the inhibition of both telomerase maintenance pathways leads to the death of Hodgkin’s lymphoma cells. Using patient-derived cell lines, we show that the cells are vulnerable to the inhibition of both pathways. The survival of the cells is impaired with either drug alone or in combination; however, the most efficient cell killing was observed during short-term treatment where telomerase inhibition (72 h) was followed by ALT inhibition (72 h). Future work will address ex vivo treatments of primary treatment-naive patient samples and investigate whether this dual treatment will impact the survival of tumor cells in other cancers.

## Data Availability

Not applicable.
